# Abnormalities of the hallux skin and nail in the course of very rare arteriovenous malformation

**DOI:** 10.1590/1677-5449.200050

**Published:** 2020-10-16

**Authors:** Marta Wasilewska, Maciej Guziński, Izabela Gosk-Bierska

**Affiliations:** 1 Wroclaw Medical University – WMU, Department of Angiology, Hypertension and Diabetology, Wroclaw, Dolnoslaskie, Poland.; 2 Wroclaw Medical University – WMU, Department of General Radiology, Interventional Radiology and Neuroradiology, Wroclaw, Dolnoslaskie, Poland.

**Keywords:** arteriovenous malformation, lower extremity, hallux, abnormal nails, skin diseases, malformação venosa, extremidade inferior, hálux, anormalidades das unhas, dermatopatias

## Abstract

Arteriovenous malformations (AVMs) are usually found in the pelvic area and the brain. These vascular anomalies are rarely reported in the toes. AVMs in the toes may be asymptomatic, but can also cause atypical symptoms. Congenital AVMs can expand as patients age and manifest in adulthood. They may be provoked by injury. Acquired AVM might be caused by iatrogenic factors, venous or arterial catheterization, percutaneous invasive vascular procedures, surgery, or degenerative vascular disorders. An AVM can damage surrounding tissues and can cause destruction of skin, nails and bones. The course of the disease is often unpredictable and diagnosis is usually delayed as a result.

## Resumo

As malformações arteriovenosas (MAVs) são geralmente encontradas na região pélvica e no cérebro.. Essas anomalias vasculares raramente são relatadas nos dedos dos pés. A MAV nesse local pode ser assintomática ou apresentar sintomas atípicos. MAVs congênitas podem evoluir com a idade e se manifestar na idade adulta. O fator provocante pode ser uma lesão traumática. Uma MAV adquirida pode ser causada por fatores iatrogênicos, cateterismo venoso e arterial, procedimentos percutâneos vasculares invasivos, cirurgias e alterações degenerativas vasculares. A MAV pode danificar tecidos adjacentes e pode causar destruição de pele, unhas e ossos. O curso da doença é muitas vezes imprevisível, e como resultado, atrasar o diagnóstico.

## INTRODUCTION

Arteriovenous malformations (AVMs) involving the foot, and especially the toes, are rarely reported.^1^^-^^3^ An AVM in this location may remain asymptomatic for a long time, or it may cause atypical symptoms. Although AVMs are usually congenital, they may not manifest until adulthood. They can be provoked by injuries. An AVM can cause a variety of abnormalities involving the skin, nails, bones, and nerves of the foot or toes that can coexist or may appear as isolated symptoms.^1^^-^^8^ The disease often has an unpredictable course and so the diagnosis is usually delayed, resulting in tissue loss or permanent deformities of the foot or toes.^1^^,^^8^

## CASE DESCRIPTION

A 48-year-old male patient with a long-term smoking history and no other significant disorders was admitted to the clinic in May 2016. His major symptoms included a 2-year history of damage to the skin and nail of the hallux of the right foot, often complicated by secondary bacterial superinfection. He complained of chronic rest pain in the big toe and swelling of the right foot but denied the typical intermittent claudication. There was no direct injury to the right foot preceding the symptoms. However, in the past the patient used to play football.

**Figure 1:**
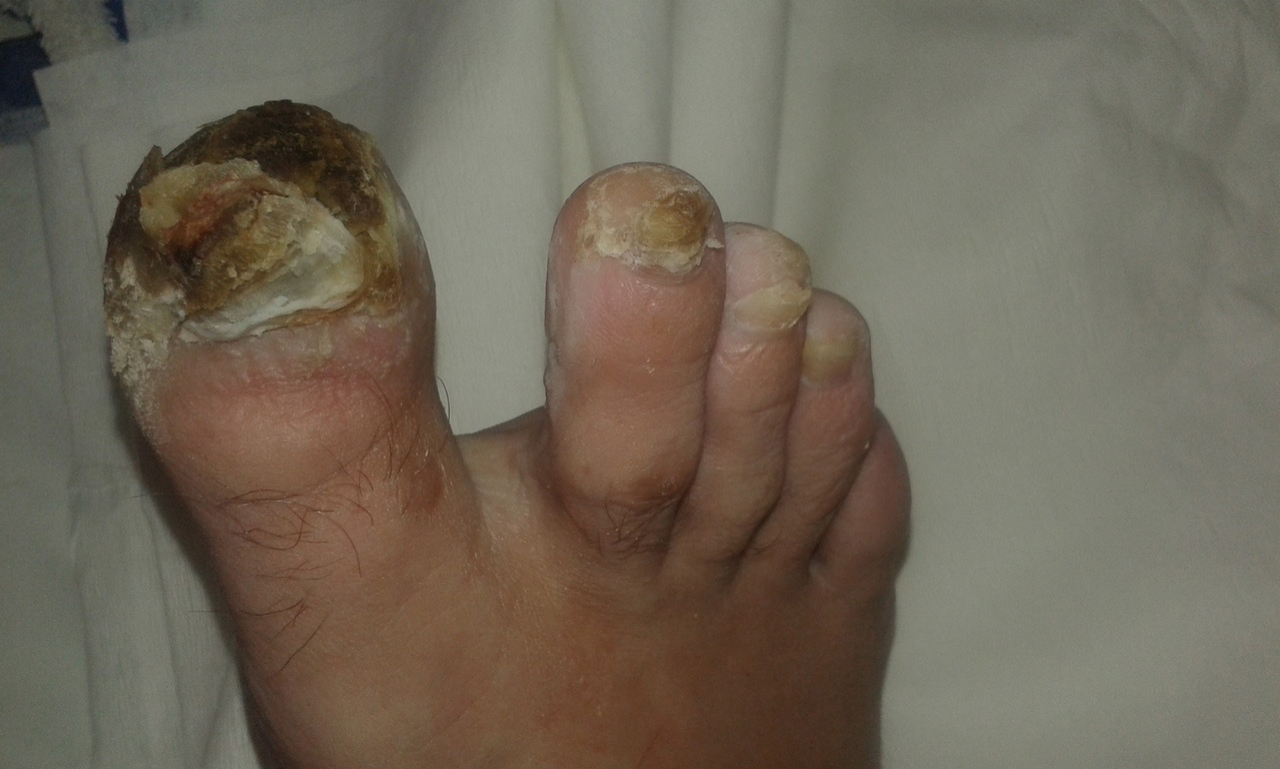
Deformation of the hallux nail plate.

**Figure 2:**
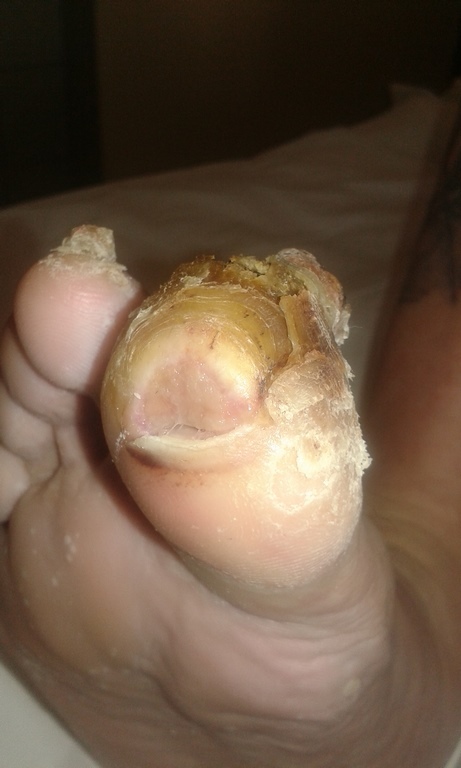
Swelling and hyperkeratosis of the hallux.

Physical examination revealed trophic changes involving the right foot, particularly advanced in the big toe and less severe in the second and third toe. The skin there was thickened with hyperkeratosis and peeling. There was also partial necrosis of the big toe. His right hallux, foot, and calf appeared swollen, with local tenderness of the great toe. The hallux nail plate was deformed, thickened, and brittle with hyperkeratosis and yellow discoloration (Figures 1 and 2). There were easily palpable symmetric pulses in both lower and upper extremities.

The only laboratory test finding was mildly elevated CRP (21 mg/l). Kidney, liver, and lipid parameters and electrolytes were all normal. Antibody tests (ANA, cANCA, pANCA, anti-CCP, RF, LA, anti-cardiolipin, and anti-beta 2 glycoprotein I antibodies) were negative. Viral tests for HBV, HCV, and HIV were negative. An oral glucose tolerance test and glycated hemoglobin assay (HbA1c 4.9%) excluded glucose metabolism disorders.

Standard imaging tests (chest X-ray, ECG, abdominal ultrasound) were normal.

**Figure 3:**
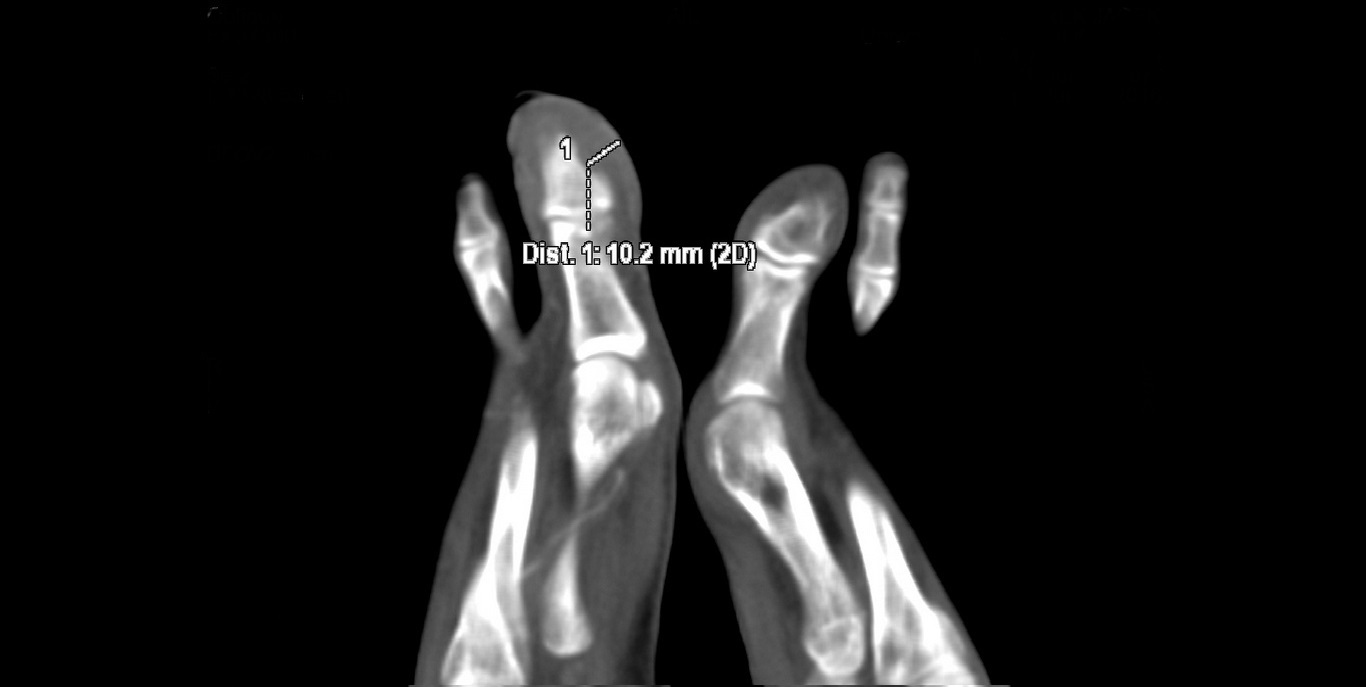
Thickening of the soft tissues of the big toe up to 10 mm.

**Figure 4:**
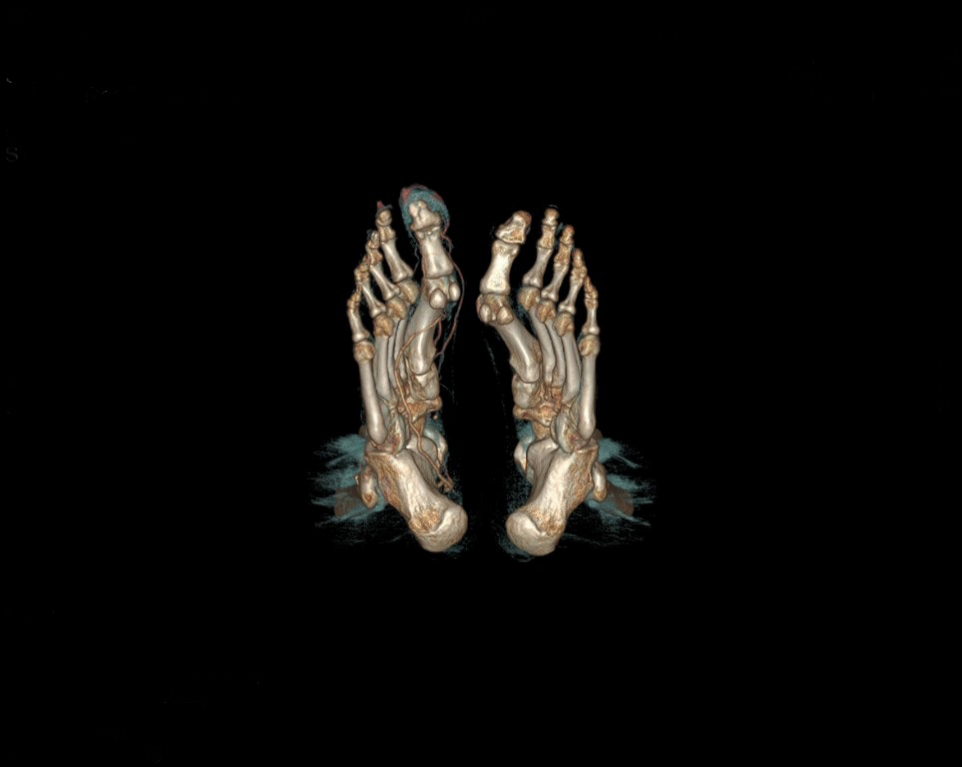
Dilated blood vessels supplying the right hallux.

**Figure 5:**
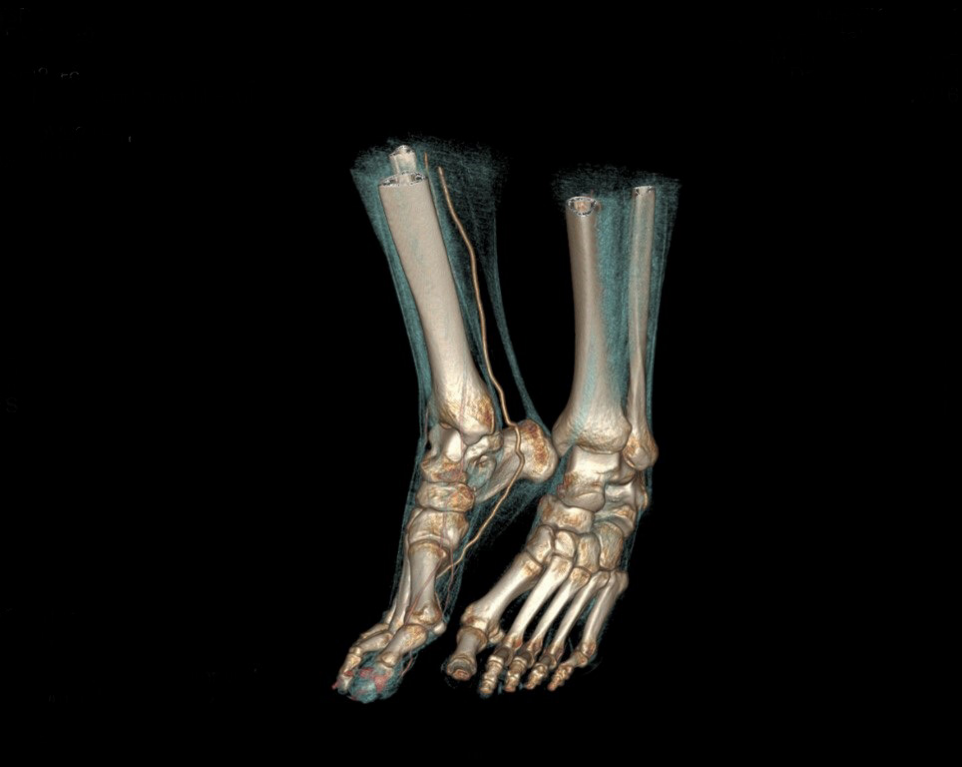
AVM of the right hallux.

X-ray of the right foot did not show any signs of destruction or inflammation involving the bones. There was no calcification in the projection of the vessels. The ankle-brachial index was normal (1.11 on the right side, 1.15 on the left). Doppler ultrasonography of the lower extremity arteries showed normal symmetrical high resistance flows. The walls of the vessels were normal. Aneurysms, which could be a potential source of peripheral embolism, were excluded. However, a slight dilatation of the right posterior tibial artery was detected. CT angiography revealed a rare vascular anomaly in the right lower extremity: hypoplasia of the distal segment of the anterior tibial artery, a slightly dilatated and tortuous posterior tibial artery, that fed the arteriovenous malformation (AVM) in the big toe with increased venous flow in the lower limb (Figures 3, 4, 5). There were no changes typical of Buerger's disease.

The decision was taken to amputate the distal phalanx of the right hallux, because of the location of the lesion, the lack of remedial possibilities, and the significantly intensified symptoms.

Histopathological examination of the removed tissues excluded neoplasm, but confirmed the features of vascular anomaly. Additionally, thickened stratum corneum with chronic inflammatory infiltration and dilated stromal vessels were described.

After the amputation, the wound healed normally, the right lower limb edema resolved, and CRP decreased in laboratory test results. At 3-year follow-up, no recurrence of skin lesions in the lower extremities was observed.

## DISCUSSION

AVMs located in the foot and toes are rarely reported. AVMs are usually innate. Congenital AVMs occur from birth and can be asymptomatic for a long time, but they can grow as patients age and manifest in adulthood.^1^^-^^3^ AVMs can also be caused by acquired factors: trauma, iatrogenic factors, catheterization, percutaneous invasive procedures, surgery, degenerative vascular changes, fragility of vessels caused by vasculitis, chronic steroid therapy, or dialysis.^1^^,^^6^^,^^8^ In view of the increasing frequency of percutaneous endovascular interventions, complications manifesting with AVMs are to be expected.^4^

AVMs in the foot can be asymptomatic, especially if they are small.

Typical symptoms are usually associated with large AVMs and can be divided into local and general. AVMs can lead to ischemia of tissues distal to the AVM, with symptoms such as intermittent claudication, coldness, pallor, or cyanosis of the skin. In more advanced cases, chronic ulcers and necrotic lesions may appear. Local venous symptoms might be caused by venous stasis and venous hypertension: swelling of the foot, calf or the whole lower extremity, pain, cutaneous redness, congestion, hyperpigmentation, dilated venous vessels, and chronic skin ulcers. General symptoms of large AVM are the result of hyperkinetic circulation and may lead to right ventricular failure.^1^^-^^4^^,^^8^

Physical examination may sometimes show a pulsatile or non-pulsatile mass, increased skin temperature, tremor of the vessel wall, and vascular murmur. AVMs can cause various skin lesions: hyperpigmentation, spots, bluish or purple nodules, port wine stains, and brownish or rusty skin discoloration, similar to chronic venous insufficiency.^1^^-^^3^ In micro-arteriovenous fistulas in the lower extremities, unusual symptoms such as refractory cellulitis resistant to standard treatment have also been described.^8^

AVMs can cause changes to nails, which are rarely reported in the literature. It is known, however, that venous stasis, venous hypertension, and also chronic limb ischemia can provoke nail changes. These abnormalities may also be the result of progressive vascular proliferation due to circulatory disorders.^1^^,^^9^^,^^10^

Nail changes or deformities are often described in chronic hemodialysis patients and may be associated with a malfunctioning arteriovenous fistula made for hemodialysis.^9^^,^^10^ These abnormalities include half and half nails, lack of lunula, brittle nails, onycholysis, leukonychia, thickened nails, increased transverse curvature of the nail plates, paronychia, hyperkeratosis, and pincer nails. For a long time, it was believed that nail changes in hemodialysis patients were related to uremia. However, it is now known that they are most likely associated with circulatory disturbances caused by arteriovenous fistulas for dialysis.^9^^,^^10^

In our case, the patient had nail changes, especially in the first and second toe. The nails were thickened, yellow, and brittle and the surrounding tissues were swollen and hypertrophied, which was probably the result of venous stasis and hypertension. Partial tissue necrosis in the great toe was the result of coexisting steal syndrome and ischemia of distal tissues.

AVMs can damage surrounding tissues, causing destruction of bone and neurological symptoms, due to compression.^1^^-^^3^^,^^6^ Isolated chronic foot pain without other symptoms such as skin changes or swelling can also be the result of AVMs, which are not usually included in the differential diagnosis for foot pain.^6^

AVMs of the lower limbs are most often diagnosed using Doppler ultrasound. However, Doppler ultrasound may not be sufficient for small lesions located distally. Therefore, if AVM is suspected, CT angiography, MRI angiography, or digital subtraction angiography should be performed.^1^^-^^8^ In clinical practice, such tests are usually considered if ischemic symptoms predominate. However, if AVMs are located distally, then symptoms of venous hypertension may prevail. This is the cause of the commonly delayed diagnosis of AVM in the foot or toes.^3^^,^^8^ In the case described, the patient had had persistent changes in the big toe and unexplained swelling of the foot for 2 years.

Surgical treatment, embolization, and sclerotherapy are the most common treatments for AVM.^1^^-^^7^ However, distal AVMs, especially in the toes, are less frequently treated interventionally because of the very small caliber of the vessels. Most often they require surgical removal, which is associated with tissue loss. Nonetheless, there are reports of effective laser treatment of AVM, but at an early stage, i.e. without coexisting necrotic changes, general symptoms, recurrent bleeding, or bone damage.

## CONCLUSION

AVMs are less common in the toes than in other regions of the body, which is the reason they are poorly understood and more difficult to diagnose. The course of AVM can be unpredictable, even with life-threatening foot tissues. The symptoms might be unusual and sometimes only isolated symptom occurs. With AVMs in peripheral locations, the symptoms of venous hypertension may predominate, while ischemia can be less extensive. In the physical examination, chronic skin changes of various types, changes in the nails, and swelling of the foot or toes, can be observed. Therefore, if these symptoms occur, AVM should be included in the differential diagnosis.

## References

[1] Yu GV, Brarens RM, Vincent AL (2004). Arteriovenous malformation of the foot: a case presentation. J Foot Ankle Surg.

[2] Lapresta A, Hermosa E, Boixeda P, Carrillo-Gijón R (2014). Malformaciones arteriovenosas digitales adquiridas: una anomalía vascular infrecuente tratada con láser. Actas Dermosifiliogr.

[3] Rustina L, Joalsen I (2017). Arteriovenous malformation in foot: surgical management combined with scleroting agent. Folia Medica Indonesiana..

[4] Mylankal KJ, Johnson B, Ettles DF (2011). Iatrogenic arteriovenous fistula as a cause for leg ulcers: a case report. Ann Vasc Dis.

[5] Selvaraj D, Stephen E, Samuel V (2019). Foot arteriovenous malformation: a singleinstitutional Experience. Indian J Vasc Endovasc Surg..

[6] Mohammad HR, Bhatti W, Pillai A (2016). An unusual presentation of arteriovenous malformation as an erosive midfoot lesion. J Surg Case Rep.

[7] Roth P, Heiss C, Koshty A, Niemann B, Boening A (2014). Posttraumatic arteriovenous fistula of the distal posterior tibial artery as cause of delayed wound healing in an unrecognized arterial injury. Thorac Cardiovasc Surg Rep.

[8] Tanaka M, Serizawa F, Nagaoka Y (2018). Two cases of micro-arteriovenous fistula in the lower extremity with misdiagnosis of refractory cellulitis. Ann Vasc Dis.

[9] Su YT, Lee JYY (2010). Pincer nail deformity associated with an arteriovenous fistula for hemodialysis. Zhonghua Pifuke Yixue Zazhi.

[10] Neild GH, Alston H, Burns A (2011). Half and half nails. NDT Plus.

